# Serum neurofilament light chain as a sensitive biomarker for neuromonitoring during extracorporeal membrane oxygenation

**DOI:** 10.1038/s41598-024-71603-z

**Published:** 2024-09-09

**Authors:** Stefanie Fischer, Lars Heubner, Stephanie May, Puya Shalchi Amirkhiz, Jens Kuhle, Pascal Benkert, Tjalf Ziemssen, Peter Spieth, Katja Akgün

**Affiliations:** 1 Center of Clinical Neuroscience, Department of Neurology, University Hospital Carl Gustav Carus, Technical University of Dresden, Fetscherstr. 74, 01307 Dresden, Germany; 2 Department of Anesthesiology, University Hospital and Faculty of Medicine Carl Gustav Carus of TU Dresden, Dresden, Germany; 3 Multiple Sclerosis Centre and Research Center for Clinical Neuroimmunology and Neuroscience (RC2NB), Departments of Biomedicine and Clinical Research, University Hospital and University of Basel, Basel, Switzerland; 4 Department of Clinical Research, Clinical Trial Unit, University Hospital Basel, University of Basel, Basel, Switzerland

**Keywords:** Extracorporeal membrane oxygenation [E04.292.451; E02.880.301], Biomarkers [D23.101], Diagnostic techniques, Neurological [E01.370.376], Prognosis, Outcomes research

## Abstract

The use of extracorporeal membrane oxygenation (ECMO) has grown rapidly, driven by the COVID-19 pandemic. Despite its widespread adoption, neurological complications pose a significant risk, impacting both mortality and survivors’ quality of life. Detecting these complications is challenging due to sedation and the heterogeneous nature of ECMO-associated neurological injury. Still, consensus of neurologic monitoring during ECMO is lacking since utilization and effectiveness of current neuromonitoring methods are limited. Especially in view of the heterogeneous nature of neurological injury during ECMO support an easily acquirable biomarker tracing neuronal damage independently from the underlying pathomechanism would be favorable. In a single-center prospective study on 34 severe acute respiratory distress syndrome (ARDS) patients undergoing ECMO, we explored the potential of serum neurofilament light chain levels (NfL) as a biomarker for neurological complications and its predictive power towards the overall outcome of ECMO patients. Individuals experiencing neurological complications (41%) demonstrated a notable rise in NfL levels (T_baseline_ median 92.95 pg/ml; T_24h_ median 132 pg/ml (IQR 88.6–924 pg/ml), p = 0.008; T_7d_ median 248 pg/ml (IQR 157–1090 pg/ml), p = 0.001). Moreover, under ECMO therapy, these patients exhibited markedly elevated concentrations compared to those without neurological complications (T_24h_ median 70.75 pg/ml (IQR 22.2–290 pg/ml), p = 0.023; T_7d_ median 128 pg/ml (IQR 51.8–244 pg/ml), p = 0.002). There was no significant difference in the NfL dynamics between surviving patients and those who died during or shortly after ECMO therapy. While NfL indicates neuro-axonal damage during intensive care with ECMO therapy, we could not identify any correlation between survival outcome and the levels of NfL, indicating that NfL may not serve as a prognostic marker for survival. Nevertheless, additional studies involving a larger patient cohort are required.

## Introduction

In 1944, the internists Kolff and Berk made the discovery that blood flowing through cellophane chambers of an artificial kidney becomes oxygenated^1^. The foundation for the development of ECMO was laid. In addition to cardiac surgery, the technique has revolutionized intensive care medicine over the past 80 years. It maintains sufficient gas exchange over days and weeks while the lung parenchyma can recover from previous damage or mature in the case of premature infants.

Despite its often life-saving effect, the use of ECMO is considered a last resort in the treatment of critically ill patients with severe impaired pulmonary function or as extracorporeal life support (ECLS) for e.g. cardiogenic shock. Primarily due to its negative side effects, particularly neurological complications and hemostatic disorders, e.g. bleeding and thrombosis, ECMO support cannot easily be recommended for every patient^2^. Distinct criteria for the eligibility of ECMO support in ARDS has been established in international guidelines^3^. ECMO support is frequently associated with neurological events such as hypoxic encephalopathy, ischemic strokes, or spontaneous intraparenchymal or extra-axial hemorrhages^4–6^. As the number of ECMO cases continues to rise, the need for standardized, sensitive, and easily applicable neuromonitoring methods becomes more urgent, especially in non-neonatal age groups. Additionally, a reliable biomarker for identifying suitable patients with a low hazard of significant neurological complications and, consequently, a low risk of mortality during ECMO support would be desirable.

A cross-disease blood-based biomarker that is highly sensitive to neuronal damage is neurofilament light chain (NfL), which has been associated with disease severity and prognosis in multiple neurological diseases and general neurodegeneration^7–9^. The neuronal cytoplasmic protein is highly expressed in large calibre myelinated axons. Its levels increase in cerebrospinal fluid (CSF) and blood proportionally to the degree of neuro-axonal damage in a variety of neurological disorders, including inflammatory, neurodegenerative, traumatic and cerebrovascular diseases. Even though changes in NfL levels in biofluids are not specific to any particular central nervous system (CNS) disease, this biomarker may have diagnostic value and significant potential in terms of prognostic assessment and disease monitoring^9^. For instance, a recent meta-analysis indicates that serum NfL shows promise as an early biomarker of neurological outcomes following cardiac arrest^10^.

With our single center prospective observational study, we aimed to examine serum neurofilament light chain (sNfL) levels, neurological complications and their potential risk factors in patients undergoing veno-venous extracorporeal membrane oxygenation (VV-ECMO) due to acute respiratory distress syndrome (ARDS). As patient recruitment took place during the Covid-19 pandemic, almost exclusively patients with ARDS secondary to COVID-19 were included. We also addressed the question whether sNfL serves as a sensitive biomarker for neurological complications during ECMO therapy and whether baseline levels prior to ECMO initiation are useful in assessing patients' mortality risk during ECMO treatment.

## Methods

### Patients and ethical approval

We conducted a single center prospective observational study, examining 34 patients at the anaesthesiological intensive care unit (ICU) at the university medical center of Dresden with severe ARDS who required VV-ECMO support in the period of 04/2021 to 11/2021. Patients were selected based on the following inclusion criteria: age ≥ 18 years, ARDS requiring VV-ECMO support, absence of pre-existing chronic or acute neurological diseases or injuries. The management of ARDS and ECMO treatment in these patients was performed according to internal guidelines and has been previously published elsewhere^11^.

The study was performed according to the Declaration of Helsinki and the study protocol was approved by the Ethics Committee of the Faculty of Medicine of the Dresden University of Technology, Germany. If patients were not able to give their informed consent, their legal representatives or an independent physician of the university clinical center received written information about the study and were asked to give written informed consent for participation considering the presumed patient`s interest. In these cases, informed consent was obtained later on, if patients were able to make decisions again.

### sNfL measurements

Serum samples were collected at three predefined different time points: just before ECMO cannulation (T_baseline_), 24 h afterwards (T_24h_) and 7 days after ECMO cannulation (T_7d_). Furthermore, patients were analyzed for demographic data (age, pre-existing diseases), routine laboratory parameters and the occurrence of complications, including clotting abnormalities, pulmonal, cardial, infectious, neurological events during or subsequent to ECMO support or exitus letalis at the ICU setting. Serum samples were stored at −80 °C till measurement. NfL levels were measured in serum using the commercially available Single Molecule Array NfL assay on a HD-1 Analyzer (SIMOA, Quanterix, Lexington, MA, USA)^12,13^. Samples were prepared as recommended in the manufacturer`s instructions (Quanterix, Lexington, MA, USA). Sample dilution was calculated and done by the instrument. The mean intra-assay coefficient of variation of duplicates was below 10%. NfL levels were measured in pg/ml and additionally log transformed for statistical analysis. Furthermore, NfL-levels were adjusted for age and BMI using a Generalized Additive Model for Location, Scale and Shape (GAMLSS model). NfL Z-scores (based on a large population of healthy controls) indicating how strongly the NfL levels deviate from levels in control persons of same age and BMI were calculated as described previously^14^.

### Statistical analysis

Our longitudinal patient data were analyzed per cohort by generalized linear mixed models for repeated measures with log link function due to right-skewed distribution pattern of the data and point of time as the fixed effect of the model. The results were adjusted for age and sex. Bonferroni correction for pairwise tests was used. Values of *p < 0.05, **p < 0.01, ***p < 0.001 and ****p < 0.0001 were considered as statistically significant.

The results are reported and graphically depicted as the median with indication of the interquartile range (IQR).

## Results

### Patient characteristics

A total of 34 patients were included in the study. The mean age was 54 years, 73.5% male and 26.5% female. Over 80% of the patients had no significant pre-existing conditions and all patients had a prehospital Barthel Index of 100. In over 90% of cases, the indication for ECMO was severe ARDS due to SARS-CoV-2 pneumonia. 44.1% survived at least until transfer to a rehabilitation facility. 41.2% of all ECMO patients developed neurological complications, of which, in turn, 57% resolved during the further course of hospitalisation. Other common complications occurring in 60–90% of patients included cardiovascular, pulmonary, coagulation, and infectious complications. All patients who deceased (55.9%) succumbed to multiorgan failure within the context of septic shock. Table 1 provides a detailed overview of the characteristics of the included patients.

**Table 1:** Characteristics of study population. ^1^Crohn’s disease, atrial fibrillation, mitral valve insufficiency, tricuspid valve insufficiency, spinal canal stenosis, chronic kidney insufficiency, renal hyperparathyroidism, cholangiocellular carcinoma; ^2^Glucocorticoids, interleukin-6 (IL6) blocking agent.

Total patient number (n)	34
Mean age, years (range)	54.2 (18–77)
Sex, n (%)	Female: 9 (26.5), male: 25 (73.5)
Severe ARDS resulting from: n (%)	
● SARS-CoV-2 pneumonia	31 (91.2)
● Pleural empyema	1 (2.9)
● Ileus with misery	1 (2.9)
● Unknown cause	1 (2.9)
Pre-existing diseases^1^, n (%)	
● Yes	6 (17.7)
● No	28 (82.3)
Survival, n (%)	
● Survived	15 (44.1)
● Deceased	19 (55.9)
Complications, n (%)	
● Neurological	14 (41.2)
● Pulmonary	26 (76.5)
● Cardiovascular	23 (67.6)
● Hemostatic	30 (88.2)
● Infectious	24 (70.6)
Co-treatment during ECMO, n (%)	
● Haemodialysis	7 (20.6)
● Catecholamines	33 (97.1)
● Coagulation products	15 (44.1)
● Red blood cell concentrates	21 (61.8)
● Platelet concentrates	3 (8.8)
● Immunomodulators^2^	14 (41.2)

### Neurological complications during ICU and ECMO

To identify potential risk factors for developing neurological adverse events during ECMO support, we compared patient characteristics of people who developed neurological complications and clinically neurologically asymptomatic patients (Table 2). There was a significant difference in the application of procoagulatory treatment (PPSB, fibrinogen) between both groups. Within the group without neurological complications 60% received procoagulatory products whereas only 21% of patients who developed neurological complications got procoagulatory therapy (p = 0.026). There were no relevant differences between the two subgroups concerning age, sex, pre-existing diseases, non-neurological complications, infusion of red cell concentrates or platelet concentrates, haemodialysis or the treatment with immune modulating drugs.

**Table 2:** Comparison of patients who developed neurological complications during VV-ECMO therapy versus patients who did not.

	Neurological complications						
	No (n = 20)			Yes (n = 14)			Chi2 test
	N	%	Clopper Pearson CI	n	%	Clopper Pearson CI	p
Age (≥ 50 years)	15	75	(50.9–91.3%)	10	71.4	(41.9–91.6%)	0.816
Sex (females)	5	25	(8.7–49.1%)	4	28.6	(8.4–58.1%)	0.816
Pre-existing diseases	4	20	(5.7–43.7%)	2	14.3	(1.8–42.8%)	0.667
Bacterial superinfection	16	80	(56.3–94.3%)	8	57.1	(28.9–82.3%)	0.150
Clotting complications	17	85	(62.1–96.8%)	13	92.9	(66.1–99.8%)	0.484
Pulmonal complications	16	80	(56.3–94.3%)	10	71.4	(41.9–91.6%)	0.562
Haemodynamic complications	13	65	(40.8–84.6%)	10	71.4	(41.9–91.6%)	0.693
Haemodialysis	4	20	(5.7–43.7%)	3	21.4	(4.7–50.8%)	0.919
Vasopressors	20	100	(83.2–100%)	13	92.9	(66.1–99.8%)	0.225
Procoagulatory treatment (PPSB, fibrinogen)	12	60	(36.1–80.9%)	3	21.4	(4.7–50.8%)	0.026
Red cell concentrates	12	60	(36.1–80.9%)	10	71.4	(41.9–91.6%)	0.493
Platelet concentrates	2	10	(1.2–31.7%)	1	7.1	(0.2–33.9%)	0.773
Immunomodulation	9	45	(23.1–68.5%)	5	35.7	(12.8–64.9%)	0.558

### sNfL levels in the ICU setting during ECMO therapy

**Fig. 1:**
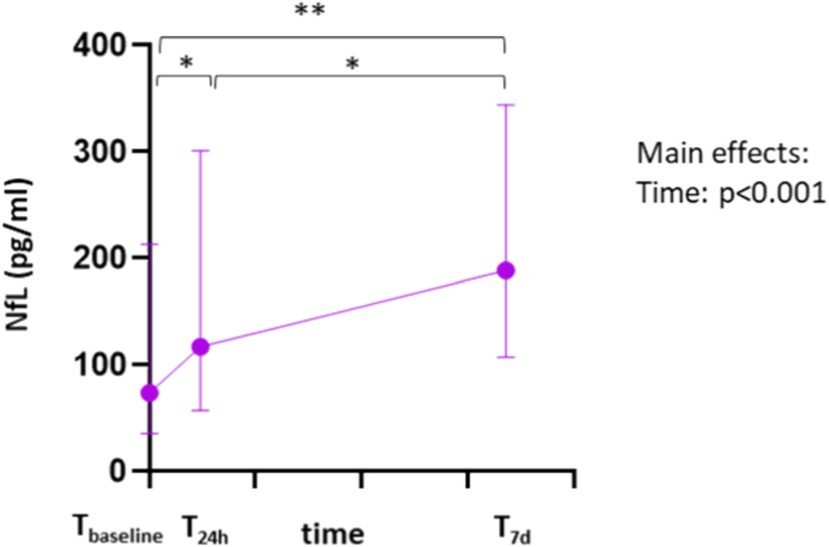
sNfL levels during ICU and ECMO therapy. Median sNfL levels with IQR in patients undergoing ECMO support just before (T_baseline_), 24 h after (T_24h_) and 7 days after ECMO cannulation (T_7d_) are presented. Asterixis indicate level of statistical significance: *p < 0.05, **p < 0.01.

An important aspect of this study was to investigate the role of sNfL in the ICU setting during ECMO therapy. First, we evaluated the overall sNfL-kinetics of all patients undergoing VV-ECMO (Fig. 1). When considering the sNfL values of all ECMO patients, it was noticeable that the values were already significantly elevated immediately before ECMO connection (T_baseline_ median 73.85 pg/ml (IQR 35.5–213 pg/ml)). 24 h after start of ECMO, a significant increase in the sNfL level was observed compared to the baseline (T_24h_ median 117 pg/ml (IQR 57.2–301 pg/ml), p = 0.011). Seven days after the start of ECMO therapy, this value showed a further significant increase (T_7d_ median 188.5 (IQR 107–344 pg/ml), p = 0.001). The sNfL values at the various measurement points in our studied patient cohort did not show any correlation with other blood parameters such as complete blood count, inflammation markers, retention parameters, cholestasis markers, or transaminases.

NfL levels are known to increase with age and decrease with increasing body mass index (BMI)^14^. Therefore, we calculated Z-scores for each sNfL value, representing the deviation of a given patient sNfL level versus same age and BMI healthy controls. Table 3 displays the Z-score distribution of all patients at time point 1 (baseline) in correlation to clinical outcome parameters (survival status, Barthel activities of daily living (ADL) index) and the occurrence of neurological complications during the course of ECMO therapy.

**Table 3:** Adjusted Z-scores of sNfL baseline levels of the study population and their distribution in the several sub cohorts.

Z-Score baseline	n (%)	Deceased n (%)	Survived n (%)	Neurological complications n (%)	No manifest neurological complications n (%)	Barthel ADL index of survivors at discharge mean (range)
< 1	2 (5.8)	1 (5.2)	1 (6.7)	1 (7.1)	1 (5)	100
≥ 1, < 2	3 (8.7)	–	3 (20)	1 (7.1)	2 (10)	21.7 (0–65)
≥ 2, < 3	7 (20.3)	4 (21)	3 (20)	1 (7.1)	6 (30)	6.7 (0–10)
≥ 3, < 4	19 (55.1)	13 (68.4)	6 (40)	9 (64.3)	10 (50)	7.5 (0–45)
≥ 4	3 (8.7)	1 (5.2)	2 (13.3)	2 (14.3)	1 (5)	15 (10–20)

8.7% of patients who underwent ECMO therapy showed sNfL values with Z-scores higher than 4 (i.e. 99.99th percentile). 50.1% of patients showed sNfL Z-scores above 3 (99.7^th^ percentile), and 20.3% above 2 (95.5th percentile). Interestingly, the sNfL values of one patient remained very low throughout the course of ECMO therapy (T_baseline_ 3.14 pg/ml, Z-score -2.2 (1.45th percentile), T_24h_ 4.46 pg/ml, Z-score -1.2 (12th percentile), T_7d_ was not collected due to early discharge from the hospital). This patient was a 37-year-old man who had an uncomplicated hospital course with rapid decannulation from ECMO treatment and a barthel index of 100 at discharge.

However, when comparing baseline sNfL Z-scores in terms of survival status, the prevalence of neurological complications, or the Barthel Index at discharge, no significant differences could be observed between the respective subgroups.

### sNfL as marker to monitor neurological complications during ICU and ECMO

One of the main aims of the study was to investigate whether sNfL is a sensitive biomarker for neurological complications during ECMO therapy and thus suitable as a monitoring marker in the ICU setting. During our study, 41% of recruited patients undergoing ECMO developed neurological complications, which manifested as intracranial parenchymal hemorrhage (11.7%), ischemic stroke (2.9%), subdural hematoma (2.9%), subarachnoidal hematoma (2.9%), posterior reversible encephalopathy syndrome (PRES) (2.9%), delirium and brain organic psychosis (11.7%), critical illness polyneuropathy and myopathy (CIP/CIM) (11.7%) and hepatic encephalopathy due to acute hepatic failure (2.9%).

**Fig. 2:**
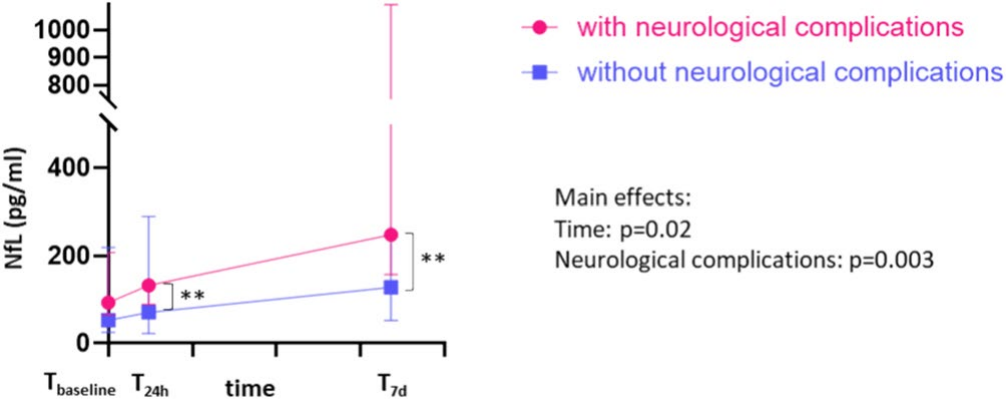
sNfL levels in patients with and without neurological complications during ICU and ECMO support. Median sNfL levels with IQR of ECMO-supported patients with neurological complications (pink) and without (purple) are presented at the points of time just before (T_baseline_), 24 h after (T_24h_) and 7 days after ECMO cannulation (T_7d_). Asterixis indicate level of statistical significance: **p < 0.01.

The course of sNfL values in relation to the occurrence of neurological complications is depicted in (Fig. 2). In comparison to patients who did not develop neurological complications during ECMO treatment, patients with neurological events showed higher sNfL values even before ECMO connection, although statistical significance had not been reached at this point in time (T_baseline (with)_ median 92.95 pg/ml (IQR 64.7–207 pg/ml) vs. T_baseline (without)_ median 52.6 pg/ml (IQR 24.5–218.5 pg/ml)). 24 h after start of ECMO therapy, the cohort of patients with neurological complications demonstrated a significant sNfL increase compared to their baseline level (T_24h (with)_ median 132 pg/ml (IQR 88.6–924 pg/ml), p = 0.008). Seven days after ECMO, the sNfL value in this subpopulation further significantly increased compared to the previous time-points (T_7d (with)_ median 248 pg/ml (IQR 157–1090 pg/ml), p = 0.001). In contrast, patients without neurological complications did not show a significant increase in sNfL within the 7-day evaluation period and presented significantly lower sNfL levels at T_24h_ (T_24h (without)_ median 70.75 pg/ml (IQR 22.2–290 pg/ml), p = 0.023) and T_7d_ (T_7d (without)_ median 128 pg/ml (IQR 51.8–244 pg/ml), p = 0.002) compared to the group with neurological complications.

### Predictive value of sNfL in terms of survival outcome

44.1% of the ECMO patients survived until discharge to a rehabilitation facility, 55.9% died within the observed time interval of seven days. Table 4 provides an overview between patients who survived and those who deceased in terms of their demographic data, received treatment measures, and the prevalence of complications during their ICU stay. Patients who passed away were on average approximately 10 years older than the survivors, had a higher prevalence of pre-existing conditions (26.3 vs. 6.7%), and experienced significantly more complications during their hospital stay.

**Table 4:** Comparison of the characteristics of patients who died and those who survived.

		Deceased	Survived
Mean age	years (age range)	58.8 (47–77)	48.4 (18–68)
Sex, female (n = 9)	n (%)	4 (44.4)	5 (55.6)
Male (n = 25)		15 (60)	10 (40)
Pre-existing diseases	n (%)	5 (26.3)	1 (6.7)
Neurological complications	n (%)	5 (26.3)	11 (60)
Pulmonary complications	n (%)	19 (100)	7 (46,7)
Cardiovascular complications	n (%)	17 (89,5)	6 (40)
Infectious complications	n (%)	19 (100)	5 (33,3)
Hemostatic complications	n (%)	18 (94.7)	12 (80)
Haemodialysis	n (%)	6 (31.6)	1 (6.7)
Catecholamine requirement	n (%)	19 (100)	14 (93.3)
Procoagulatory therapy	n (%)	11 (57.9)	4 (26.7)
Red blood cell concentrates	n (%)	15 (78.9)	7 (46.7)
Platelet concentrates	n (%)	2 (10.5)	1 (6.7)

**Fig. 3:**
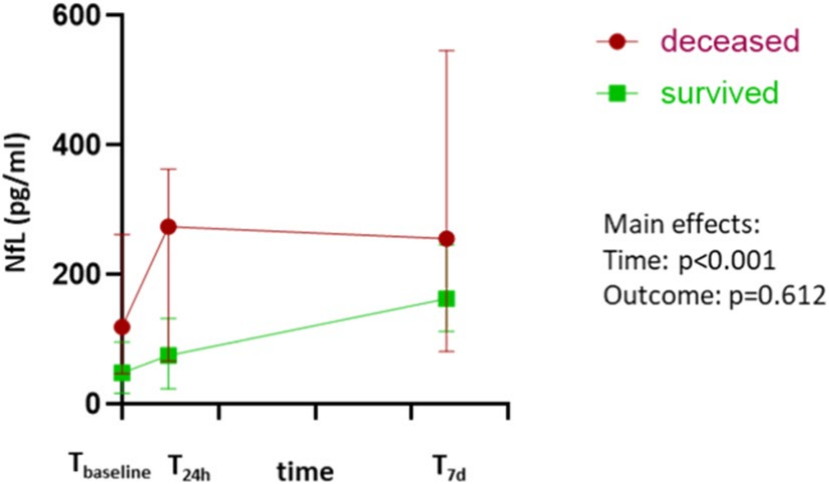
sNfl levels depending on survival during ICU and ECMO. Comparison between median sNfL levels of ECMO-supported patients depending on their outcomes (survived green; deceased red) at the points of time just before (T_baseline_), 24 h after (T_24h_) and 7 days after ECMO cannulation (T_7d_).

The course of NfL values in relation to survival status is depicted in (Fig. 3). When considering baseline NfL levels (T_baseline_), there was no significant difference between patients who survived (T_baseline (survived)_ median 48.3 pg/ml (IQR 16.8–95.8 pg/ml)) and those who passed away during the subsequent course of intensive medical treatment (T_baseline (deceased)_ median 119 pg/ml (IQR 47,4–262 pg/ml), p = 0.45). Furthermore, no significant difference was observed between the two subgroups at 24 h and 7 days after ECMO initiation in the subsequent course of treatment, although patients with a fatal outcome tended to exhibit higher values compared to survivors (T_24h (deceased)_ median 274 pg/ml (IQR 66.3–363 pg/ml) vs. T_24h (survived)_ median 75.2 pg/ml (IQR 23.5–132 pg/ml); T_7d (deceased)_ median 255.5 pg/ml (IQR 81.1–546 pg/ml) vs. T_7d (survived)_ median 162.5 pg/ml (IQR 112–246 pg/ml)).

## Discussion

ICU treatment and ECMO therapy are associated with an increased risk of developing neurological complications. However, sensitive and specific biomarkers that allow for comprehensive monitoring of sedated patients regarding neurological complications are not yet standardized. Furthermore, reliable biomarkers are lacking to identify patients at risk for neurological complications as well as an unfavorable clinical course under planned intensive care measures. During the Covid-19 pandemic, the examined cohort almost exclusively comprised patients with Covid-19-associated severe ARDS, with Covid-19 infection itself already proven to increase the risk of neurological complications.

NfL is a sensitive biomarker detecting neuronal damage in several neurological diseases, neurodegeneration and neurotrauma and seems promising for detecting neurological complications during ECMO therapy^8,15–19^. Furthermore, NfL serves as potential prognostic marker in various diseases, as it is considered a predictor of disease activity in patients with multiple sclerosis (MS), as an early biomarker of neurological outcomes following cardiac arrest, as well as marker for cognitive worsening in Alzheimer`s disease (AD), frontotemporal dementia (FTD) and parkinson`s disease (PD), and of motor worsening in patients with amyotrophic lateral sclerosis (ALS) and atypical parkinsonian disorders (APDs)^7,10^.

As people are getting older and present more comorbidities a reliable biomarker predicting the outcome of patients undergoing ECMO support is preferable to screen for those who benefit from this invasive treatment. However, in our study increased baseline sNfL levels were not associated with a poor prognosis comparing survivors and patients who died during or shortly after ECMO therapy. Furthermore, sNfL levels before ECMO initiation could not predict the risk for subsequent neurological complications during ECMO therapy. Although patients who developed neurological complications during ECMO had, on average, higher sNfL values before ECMO initiation compared to patients without neurological symptoms later on, this difference did not reach statistical significance. Our results are in line with those of Smeele et al. who observed that sNfL levels of COVID-19 patients neither at ICU admission nor at their peak predict 90 days-mortality^6^. However, available research data are not consistent in respect to this question as a previous study could demonstrate an association between sNfL and mortality in ICU patients^8,20^.

Interestingly, the baseline sNfL levels before ECMO connection were elevated in nearly all patients, regardless of their subsequent clinical course, when compared to age-matched healthy controls by calculating Z-scores. In this context, more than 80% of the patients had their baseline sNfL values exceeding the 95.5th percentile versus healthy controls (i.e. versus 4.5% of same age and BMI controls expected to show higher sNfL levels). Possible explanatory approaches for the pre-existing elevated sNfL levels observed in nearly all patients include preceding and persistent respiratory insufficiency, e.g. hypoxemia^21^, reduced venous CNS drainage due to elevates ventilator pressures^22,23^, negative effects of long-term sedation and analgesia^24,25^ as well as neurotoxicity due to Covid-associated systemic inflammatory response^20,26–28^.

Regardless of the clinical outcome, the dynamics of sNfL values during the course of ICU treatment exhibited an upward trend in all patients, even in those who did not manifest neurological complications. Therefore, it must be assumed that at least subclinical neurological damage occurred. Several sources and pathomechanisms for (subclinical) neurological injury during intensive care treatment have to be considered: Once again, it must be noted that almost all patients of our study were infected with COVID-19. Previous studies could demonstrate that severe COVID-19 infection itself leads to a sNfL increase in a non-linear fashion in the first month after ICU admission and is associated with higher sNfL compared with non-COVID-19 ICU controls^8,20,27^. NfL increase in critically ill COVID-19 patients does not surprise considering previously discovered pathomechanisms like toxic or metabolic encephalopathy^8,28^, axonal damage due to inflammatory response^8,29^, endothelial dysregulation due to viral entry^3^ as well as cytokine storm with associated prothrombotic state^3^. Furthermore, ECMO application holds high risk for neurological damage, among others due to rapid correction of hypercarbia^30^, severe hemostatic disorders as acquired von Willebrand syndrome due to higher sheer stress of the ECMO circuit, aggressive anticoagulation, thrombocytopenia and thrombosis^6,31,32^. Though not all patients receiving ECMO support develop clinically apparent neurological symptoms, Marinoni et al. could show that cerebral microemboli are detectable in neurologically asymptomatic ECMO patients as well^33^ indicating at least one source for subclinical neuronal damage. In addition, during our study ICU-associated complications like bacterial superinfections occurred frequently in the recruited study population, presumably leading to systemic inflammatory response syndrome, which could further contribute to brain injury^34^. Based on the collected sNfL data it can be assumed, that patients undergoing ECMO support sustain at least subclinical neuronal damage, emphasizing the compelling need for sensitive neuromonitoring in order to initiate further and targeted diagnostics in case of abnormalities.

Even though none of the patients in our cohort succumbed primarily to their neurological complications, sensitive and early detection of neurological complications can be live saving. On the other hand, a reliable neuromonitoring method with the early identification of neurological complications could contribute to early adjustment of therapeutic goals in case of predicted severe neurological disability. While sNfL values before the initiation of ECMO therapy do not appear to serve as predictors for the subsequent clinical course under ECMO support, our data does reveal significantly elevated sNfL levels in patients with manifest neurological complications acquired during the course of ECMO support independent on their sources compared to patients developing no evident neurological injury.

Molecular biomarkers like sNfL are of significant relevance in addition to clinical examination especially in ICU patients that are mostly sedated and often treated with muscle relaxants. Several methods of neuromonitoring during ECMO have been analyzed, but its use is limited due to inconsistency because of different methods, high costs, limited availability of trained staff and the lack of validation in large-scale studies^35^. Furthermore, many of these assessed monitoring methods depend on a specific pathophysiology or epiphenomenon (e.g. elevated or reduced cerebral excitability, cortical desoxygenation etc.) which leads to a decreased sensitivity of detecting neuronal damage, particularly located in deep brain structures or due to other underlying factors.

Especially with respect to the broad spectrum of possible neurological complications during ECMO support, a monitoring method detecting neuronal damage instead of a single possible pathomechanism will contribute to a necessarily high sensitivity. Considering this, blood based neuronal biomarkers are promising candidates of a future standardized neuromonitoring protocol for patients undergoing ECMO support. There are published reports of the evaluation of other blood-based parameters like NSE, S100b and GFAP with regard to their eligibility for neuromonitoring during ECMO^35–39^. However, despite their demonstrated significant association with acute brain injury, validated biomarker-based monitoring protocols applicable for ECMO therapy are still lacking. Furthermore, most studies regarding these parameters were conducted speciously on biomarker dynamics in infants undergoing ECMO. Reasons, why sNfL should be part of single or multi-panel blood biomarkers include its eligibility to detect axonal damage directly (vs. GFAP as glial parameter), the requirement of small blood volumes for assay, fast processing, precise quantification, low cost, minimal technical expertise required for the assay and the intense and ongoing research on sNfL, including studies examining potential neuroprotective agents as well^40^. Furthermore, it becomes apparent, that the combination of different neuronal biomarkers, including sNfL, could contribute to etiological classification of neuronal damage which could prospectively be helpful in an ICU setting as well^41^.

Regarding potential risk factors for the development of neurological complications in our cohort, we observed an association with the absence of administration of procoagulatory products. Due to the small size of our study population and the study design, no causal relationship can be inferred from our data. However, given that intracranial hemorrhages are a leading complication in COVID-19-infected patients under ECMO support, future studies investigating this relationship more closely could be promising^3^.

Some limitations of our study have to be discussed including the relatively small sample size and the lack of routine imaging, electrophysiologic measurements and consistent neurological scoring (e.g. GCS, NIHSS) to assign degree and kind of selected neurological complications more in detail. Furthermore, data on long-term clinical outcome of patients who survived are missing. The current project was planned as a pilot study in our anesthesiology-led intensive care unit. Here, acute neurological diagnostic procedures were performed as needed in the event of clinical signs of deterioration (GCS deterioration, pupillary difference, etc.). A standardized collection of functional neurological test parameters at the respective time points, particularly at discharge or transfer, was not initially planned in the study design. However, it must be consistently considered in future projects to investigate the association with brain-centered outcomes. A corresponding follow-up project is already in planning, which will include the mentioned implementation of standardized neurological diagnostics as well as a larger sample size. One of the main reasons for ending recruitment for the current study was the alignment with the sample sizes of previously published ECMO studies, coupled with the decline of the COVID-19 pandemic, which made further patient recruitment difficult.

As a final limiting factor of this study, we would like to point out the still not yet firmly established neurofilament reference ranges. In the literature, a cutoff of 10 pg/ml is mentioned, which is also used for other neurological diseases. It is assumed that neurofilament levels are likely normal if the value is below 10 pg/ml. Even for the assessment of BMI- and age-adjusted Z-scores, there are currently only reference values for previously studied neurological conditions. For example, in multiple sclerosis, a significant increase in NfL is present at a Z-score of 1.25, and a secure neurodestruction is assumed at a value of 1.5^14^. To illustrate the neurofilament dynamics at different sampling times, we deliberately chose absolute neurofilament values to avoid information loss through Z-score transformation.

Nevertheless, the availability of a biomarker for neurological monitoring of ICU patients, especially patients undergoing ECMO support, could be of significant relevance concerning clinical decision-making. Our results show that significantly increasing sNfL levels during ECMO therapy indicate neurological complications, and suggest sNfL levels as potential molecular biomarker as part of a sensitive neuro-monitoring tool to predict neuro-axonal damage in ICU setting. Further studies are essential to define distinct sNfL levels and the importance of sNfL variation over time, to estimate the potential of sNfL measurements to predict neurological complications in detail and to weigh mortality risks and clinical outcome in ICU patients.

## Data Availability

All data generated during/or analysed during this study are available from the corresponding author on reasonable request.

## Abbreviations

AD: Alzheimer’s disease
ALS: Amyotrophic lateral sclerosis
APDs: Atypical parkinsonian disorders
ARDS: Acute respiratory distress syndrome
CI: Confidence interval
CIP/CIM: Critical illness myopathy/critical illness polyneuropathy
CNS: Central nervous system
CSF: Cerebrospinal fluid
ECMO: Extracorporeal membrane oxygenation
Etc.: Et cetera
FTD: Frontotemporal dementia
GFAP: Glial fibrillary acidic protein
ICH: Intracranial hemorrhage
ICU: Intensive care unit
IQR: Interquartile range
MS: Multiple sclerosis
NfL: Neurofilament light chain
ns: Not significant
NSE: Neuron specific enolase
PD: Parkinson: s disease
PRES: Posterior reversible encephalopathy syndrome
SAH: Subarachnoidal hemorrhage
SDH: Subdural hemorrhage
sNfL: Neurofilament light chain in serum
vs.: Versus
VV-ECMO: Veno-venous extracorporeal membrane oxygenation

## References

[1] Kolff, W. J. The artificial kidney. *Am. Pract. Dig. Treat.* **13**, 219–220 (1962). 14458011

[2] Mirus, M. *et al.* Hemostatic disorders associated with extracorporeal membrane oxygenation. *Minerva Anestesiol.* **89**(6), 586–596 (2023). 37283541 10.23736/S0375-9393.23.17121-5

[3] Combes, A. *et al.* Extracorporeal membrane oxygenation for severe acute respiratory distress syndrome. *N. Engl. J. Med.* **378**(21), 1965–1975 (2018). 29791822 10.1056/NEJMoa1800385

[4] Migdady, I. *et al.* Brain injury and neurologic outcome in patients undergoing extracorporeal cardiopulmonary resuscitation: A systematic review and meta-analysis. *Crit. Care Med.* **48**(7), e611–e619 (2020). 32332280 10.1097/CCM.0000000000004377

[5] Shoskes, A. *et al.* Brain injury is more common in venoarterial extracorporeal membrane oxygenation than venovenous extracorporeal membrane oxygenation: A systematic review and meta-analysis. *Crit. Care Med.* **48**(12), 1799–1808 (2020). 33031150 10.1097/CCM.0000000000004618

[6] Kannapadi, N. V. *et al.* Neurological complications in COVID-19 patients with ECMO support: A systematic review and meta-analysis. *Heart Lung Circ.* **31**(2), 292–298 (2022). 34756659 10.1016/j.hlc.2021.10.007 PMC8553269

[7] Khalil, M. *et al.* Neurofilaments as biomarkers in neurological disorders. *Nat. Rev. Neurol.* **14**(10), 577–589 (2018). 30171200 10.1038/s41582-018-0058-z

[8] Smeele, P. J. *et al.* Neurofilament light increases over time in severe COVID-19 and is associated with delirium. *Brain Commun.* **4**(4), fcac195 (2022). 35938070 10.1093/braincomms/fcac195 PMC9351727

[9] Gaetani, L. *et al.* Neurofilament light chain as a biomarker in neurological disorders. *J. Neurol. Neurosurg. Psychiatr.* **90**(8), 870–881 (2019). 10.1136/jnnp-2018-320106 30967444

[10] Wang, S. L. *et al.* Serum neurofilament light chain as a predictive marker of neurologic outcome after cardiac arrest: A meta-analysis. *BMC Cardiovasc. Disord.* **23**(1), 193 (2023). 37061702 10.1186/s12872-023-03220-z PMC10105388

[11] Heubner, L. *et al.* Extreme obesity is a strong predictor for in-hospital mortality and the prevalence of long-COVID in severe COVID-19 patients with acute respiratory distress syndrome. *Sci. Rep.* **12**(1), 18418 (2022). 36319681 10.1038/s41598-022-22107-1 PMC9626466

[12] Disanto, G. *et al.* Serum neurofilament light: A biomarker of neuronal damage in multiple sclerosis. *Ann. Neurol.* **81**(6), 857–870 (2017). 28512753 10.1002/ana.24954 PMC5519945

[13] Wilson, D. H. *et al.* The simoa HD-1 analyzer: A novel fully automated digital immunoassay analyzer with single-molecule sensitivity and multiplexing. *J. Lab. Autom.* **21**(4), 533–547 (2016). 26077162 10.1177/2211068215589580

[14] Benkert, P. *et al.* Serum neurofilament light chain for individual prognostication of disease activity in people with multiple sclerosis: A retrospective modelling and validation study. *Lancet Neurol.* **21**(3), 246–257 (2022). 35182510 10.1016/S1474-4422(22)00009-6

[15] Moseby-Knappe, M. *et al.* Serum neurofilament light chain for prognosis of outcome after cardiac arrest. *JAMA Neurol.* **76**(1), 64–71 (2019). 30383090 10.1001/jamaneurol.2018.3223 PMC6440255

[16] Rahmig, J. *et al.* Serum neurofilament light chain levels are associated with stroke severity and functional outcome in patients undergoing endovascular therapy for large vessel occlusion. *J. Neurol. Sci.* **429**, 118063 (2021). 34488043 10.1016/j.jns.2021.118063

[17] Rana, O. R. *et al.* Neurofilament light chain as an early and sensitive predictor of long-term neurological outcome in patients after cardiac arrest. *Int. J. Cardiol.* **168**(2), 1322–1327 (2013). 23287695 10.1016/j.ijcard.2012.12.016

[18] Tiedt, S. *et al.* Serum neurofilament light: A biomarker of neuroaxonal injury after ischemic stroke. *Neurology* **91**(14), e1338–e1347 (2018). 30217937 10.1212/WNL.0000000000006282

[19] Wihersaari, L. *et al.* Neurofilament light as an outcome predictor after cardiac arrest: A post hoc analysis of the COMACARE trial. *Intensive Care Med.* **47**(1), 39–48 (2021). 32852582 10.1007/s00134-020-06218-9 PMC7782453

[20] Sutter, R. *et al.* Serum neurofilament light chain levels in the intensive care unit: Comparison between severely ill patients with and without coronavirus disease 2019. *Ann. Neurol.* **89**(3), 610–616 (2021). 33377539 10.1002/ana.26004

[21] Kyng, K. J. *et al.* Neurofilament light chain serum levels after hypoxia-ischemia in a newborn piglet model. *Front. Pediatr.* **10**, 1068380 (2022). 36699314 10.3389/fped.2022.1068380 PMC9869944

[22] Traub, J. *et al.* Serum phosphorylated tau protein 181 and neurofilament light chain in cognitively impaired heart failure patients. *Alzheimers Res. Ther.* **14**(1), 149 (2022). 36217177 10.1186/s13195-022-01087-4 PMC9549648

[23] Taran, S., Wahlster, S. & Robba, C. Ventilatory targets following brain injury. *Curr. Opin. Crit. Care* **29**(2), 41–49 (2023). 36762685 10.1097/MCC.0000000000001018

[24] Evered, L. *et al.* Association of changes in plasma neurofilament light and tau levels with anesthesia and surgery: Results from the CAPACITY and ARCADIAN studies. *JAMA Neurol.* **75**(5), 542–547 (2018). 29459944 10.1001/jamaneurol.2017.4913 PMC5885271

[25] Hou, Y. R. *et al.* Effect of dexmedetomidine on postoperative plasma neurofilament light chain in elderly patients undergoing thoracoscopic surgery: A prospective randomized controlled trial. *Clin. Interv. Aging* **18**, 1565–1576 (2023). 37727450 10.2147/CIA.S422560 PMC10506605

[26] Amit, M. *et al.* Loss of p53 drives neuron reprogramming in head and neck cancer. *Nature* **578**(7795), 449–454 (2020). 32051587 10.1038/s41586-020-1996-3 PMC9723538

[27] Hay, M. *et al.* Serum neurofilament light is elevated in COVID-19 positive adults in the ICU and is associated with co-morbid cardiovascular disease, neurological complications, and acuity of illness. *Cardiol. Cardiovasc. Med.* **5**(5), 551–565 (2021). 34708189 10.26502/fccm.92920221 PMC8547787

[28] Frontera, J. A. *et al.* A prospective study of neurologic disorders in hospitalized patients with COVID-19 in New York city. *Neurology* **96**(4), e575–e586 (2021). 33020166 10.1212/WNL.0000000000010979 PMC7905791

[29] Del Valle, D. M. *et al.* An inflammatory cytokine signature predicts COVID-19 severity and survival. *Nat. Med.* **26**(10), 1636–1643 (2020). 32839624 10.1038/s41591-020-1051-9 PMC7869028

[30] Xie, A. *et al.* Neurologic complications of extracorporeal membrane oxygenation: A review. *J. Cardiothorac. Vasc. Anesth.* **31**(5), 1836–1846 (2017). 28625752 10.1053/j.jvca.2017.03.001

[31] Kalbhenn, J. *et al.* Acquired von willebrand syndrome and impaired platelet function during venovenous extracorporeal membrane oxygenation: Rapid onset and fast recovery. *J. Heart Lung Transplant.* **37**(8), 985–991 (2018). 29650295 10.1016/j.healun.2018.03.013

[32] Uriel, N. *et al.* Acquired von willebrand syndrome after continuous-flow mechanical device support contributes to a high prevalence of bleeding during long-term support and at the time of transplantation. *J. Am. Coll. Cardiol.* **56**(15), 1207–1213 (2010). 20598466 10.1016/j.jacc.2010.05.016

[33] Marinoni, M. *et al.* Cerebral microemboli detected by transcranial doppler in patients treated with extracorporeal membrane oxygenation. *Acta Anaesthesiol. Scand.* **60**(7), 934–944 (2016). 27109305 10.1111/aas.12736

[34] Xiang, Y. *et al.* Inflammatory mechanisms involved in brain injury following cardiac arrest and cardiopulmonary resuscitation. *Biomed. Rep.* **5**(1), 11–17 (2016). 27330748 10.3892/br.2016.677 PMC4906809

[35] Bembea, M. M. *et al.* Neuromonitoring during extracorporeal membrane oxygenation: A systematic review of the literature. *Pediatr. Crit. Care Med.* **16**(6), 558–564 (2015). 25828783 10.1097/PCC.0000000000000415

[36] Bembea, M. M. *et al.* Glial fibrillary acidic protein as a brain injury biomarker in children undergoing extracorporeal membrane oxygenation. *Pediatr. Crit. Care Med.* **12**(5), 572–579 (2011). 21057367 10.1097/PCC.0b013e3181fe3ec7 PMC3686089

[37] Floerchinger, B. *et al.* Neuron-specific enolase serum levels predict severe neuronal injury after extracorporeal life support in resuscitation. *Eur. J. Cardiothorac. Surg.* **45**(3), 496–501 (2014). 23878016 10.1093/ejcts/ezt370

[38] Gazzolo, D. *et al.* Elevated S100B protein as an early indicator of intracranial haemorrhage in infants subjected to extracorporeal membrane oxygenation. *Acta Paediatr.* **91**(2), 218–221 (2002). 11952012 10.1111/j.1651-2227.2002.tb01698.x

[39] Nguyen, D. N. *et al.* Serum S100B protein could help to detect cerebral complications associated with extracorporeal membrane oxygenation (ECMO). *Neurocrit. Care* **20**(3), 367–374 (2014). 23860667 10.1007/s12028-013-9874-6

[40] Aharoni, R. *et al.* Neuroprotective effect of glatiramer acetate on neurofilament light chain leakage and glutamate excess in an animal model of multiple sclerosis. *Int. J. Mol. Sci.* **22**(24), 13419 (2021). 34948217 10.3390/ijms222413419 PMC8707261

[41] Zhu, N. *et al.* Plasma glial fibrillary acidic protein and neurofilament light chain for the diagnostic and prognostic evaluation of frontotemporal dementia. *Transl. Neurodegener.* **10**(1), 50 (2021). 34893073 10.1186/s40035-021-00275-w PMC8662866

